# Molecular epidemiology and genetic characterization of SARS-CoV-2 in Kuwait: A descriptive study

**DOI:** 10.3389/fmicb.2022.858770

**Published:** 2022-08-26

**Authors:** Nada Madi, Hussain A. Safar, Abu Salim Mustafa, Wassim Chehadeh, Mohammad Asadzadeh, Mohammed Sadeq, Ebaa Alawadhi, Ali Al-Muhaini, Fahad A. Benthani

**Affiliations:** ^1^Department of Microbiology, Faculty of Medicine, Kuwait University, Kuwait, Kuwait; ^2^OMICS Research Unit, Faculty of Medicine, Kuwait University, Kuwait, Kuwait; ^3^Jaber Al-Ahmad Armed Forces Hospital, Kuwait, Kuwait; ^4^Jaber Al-Ahmad Hospital, Ministry of Health, Kuwait, Kuwait; ^5^Research Institute for Developmental Medicine, Johannes Kepler University of Linz, Linz, Austria

**Keywords:** molecular epidemiology, SARS-CoV-2, Nanopore sequencing technology, Kuwait, variants

## Abstract

Severe acute respiratory syndrome coronavirus-2 (SARS-CoV-2) has been fatal to human health, affecting almost the entire world. Here we reported, for the first time, characterization of the genetic variants of SARS-CoV-2 circulating in Kuwait to understand their genetic diversity and monitor the accumulation of mutations over time. This study randomly enrolled 209 COVID-19 patients whose nasopharyngeal swabs were positive for SARS-CoV-2 between February 2020 and June 2021 using RT-PCR. The whole genomes of SARS-CoV-2 from the nasopharyngeal swabs were sequenced using the Oxford Nanopore sequencing technology following the ARTIC network protocol. Whole-genome sequencing has identified different clades/sub-clades circulating in Kuwait, mimicking the virus’s global spread. Clade 20A was dominant from February 2020 until January 2021, and then clade 20I (Alpha, V1) emerged and dominated. In June 2021, the number of cases infected with clades 21I, 21A, and 21 J (Delta) increased and dominated. We detected several known clade-defining missense and synonymous mutations and other missense mutations in the genes encoding important viral proteins, including ORF1a, S, ORF3a, ORF8 regions and a novel mutation in the N region. ORF1ab region harbored more mutations and deletions (*n* = 62, 49.2%) compared to the other 12 gene regions, and the most prevalent missense mutations were P314L (97%) in ORF1b and D614G (97%) in the S glycoprotein regions. Detecting and analyzing mutations and monitoring the evolution of SARS-CoV-2 over time is essential to help better understand the spread of various clades/strains of SARS-CoV-2 and their implications for pathogenesis. In addition, knowledge of the circulating variants and genome sequence variability of SARS-CoV-2 may potentially influence the development of vaccines and antiviral drugs to control the COVID-19 pandemic.

## Introduction

The coronavirus disease 2019 (COVID-19) epidemic was caused by a novel coronavirus first identified in December 2019 in Wuhan, China, and now has extended worldwide and turned into a global pandemic (Huang et al., 2020). COVID-19 was rapidly caused by a coronavirus named severe acute respiratory syndrome coronavirus 2 (SARS-CoV-2; Zhu et al., 2020). The virus belongs to the β coronavirus family, including other coronaviruses capable of infecting humans; four coronaviruses (229E, NL63, OC43, and HKU1) cause mild symptoms of the common cold. However, the other two, SARS-CoV and MERS-CoV, can cause severe symptoms and eventually death, with 10 and 37% fatalities, respectively (Gorbalenya et al., 2020; Mohammad Lokman et al., 2020). Although the case fatality rate of SARS-CoV-2 is estimated to be around 3.4%,^1^ which is lower than that of SARS-CoV and MERS-CoV, nevertheless, effective treatment and control are essential as the global toll has already surpassed 5 million confirmed deaths.^2^ The pandemic has significantly impacted social and economic activity worldwide.

SARS-CoV-2 is a positive-sense, single-stranded RNA virus with a genome approximately 30 kb in size. The genome of SARS-CoV-2 encodes for nonstructural proteins (NSPs) and structural proteins in different open reading frames (ORFs; Laamarti et al., 2020; Wu et al., 2020). The polyproteins pp1a and pp1ab are encoded by ORFs 1a and 1b at the 5′-end, covers more than two-thirds of the total genome size and encodes 16 nonstructural proteins (numbered from NSP1 to NSP16). In contrast, the 3′-end of the genome (21–29 kb) encodes for six accessory proteins named ORF3a (presumed apoptotic factor), ORF6 (presumed IFN-1 antagonist), ORF7a (presumed leukocyte modulator), ORF7b, ORF8 (presumed 62 immunomodulators), and ORF10 (unknown function) along with four structural proteins: spike glycoprotein (S), an envelope protein (E), membrane glycoprotein (M), and nucleocapsid protein (N). Genetic variation in several viral proteins, such as RNA-dependent RNA polymerase and spike glycoprotein, which are vital drug targets, impacts the efficacy of current vaccines and antiviral treatments (Miao et al., 2021). Therefore, detecting and analyzing mutations and monitoring the evolution of SARS-CoV-2 over time is essential to our understanding of SARS-CoV-2. In addition, knowledge of the circulating variants and genome sequence variability of SARS-CoV-2 may potentially impact the development of vaccines and antiviral drugs to control the COVID-19 pandemic (Andersen et al., 2020). Furthermore, SARS-CoV-2 genetic variation (Volz et al., 2021) and host genetics influence the pathogenesis of the virus and eventually affect infection and mortality rates (Wang et al., 2020).

In Kuwait, the first cases of SARS-CoV-2 were documented in February 2020 in travellers arriving from Iran. After that, more cases were imported from other parts of the world, mainly Europe (Italy), until March 2020, when most air traffic was suspended. In March 2020, the rapidly growing number of confirmed cases alarmed the government, which decided to implement many restrictions to decrease the spread of SARS-CoV-2 into and inside the country. The number of cases steadily increased from 2020 until July 2021, when the number of cases gradually decreased after introducing an intensive COVID-19 vaccination program. As of January 12 2022, when this paper was written, 437,602 confirmed cases and 2,472 deaths had been recorded in Kuwait.^3^ Since there is a large gap in the genomic characterization of SARS-CoV-2 circulating in the Kuwaiti population, this study aimed to characterize the genetic variants in 209 SARS-CoV-2 complete genomes from infected patients in Kuwait for a detailed understanding of their genetic diversity and to monitor the accumulation of mutations over time. Since one of the major viral antigens, spike glycoprotein is in the current vaccine, it is important to know whether mutations in this protein facilitate escape from host antibodies, potentially compromising vaccine efficiency. Currently, the selection of a variant in a population is perhaps not determined by host antibody because there is no adequate immunity in the individuals to systematically push the virus in a given direction (Korber et al., 2020). In contrast, the variant could rapidly outcompete and replace other circulating variants if variant mutations in the spike upsurge transmissibility (Korber et al., 2020). Therefore, awareness of the circulating variants and genome sequence variability of SARS-CoV-2 in Kuwait may influence vaccines and antiviral drug development to control the COVID-19 pandemic.

## Materials and methods

### Study population and data collection

This study randomly selected and enrolled nasopharyngeal swabs from 209 patients identified as SARS-CoV-2 positive between February 2020 and June 2021. The samples were retrieved from the virology labs at two hospitals in Kuwait—Jaber Al-Ahmad Hospital and Mubarak Al-Kabeer Hospital—and subjected to Real-Time Reverse Transcriptase PCR assays (RT-PCR) for the detection of SARS-CoV-2, as per the Ministry of Health guidelines in Kuwait. Samples with low viral load (Ct > 35) were excluded from the study population. Data from the patient’s demography (age, gender, and nationality) and RT-PCR results for SARS-CoV-2 viral nucleic acid detection were retrieved from the electronic medical record system. The patients were classified into five categories: (1) asymptomatic: individuals who test positive for SARS-CoV-2 but have no symptoms. (2) Mild illness: individuals with any of several signs and symptoms (e.g., fever, cough, sore throat, malaise, headache, muscle pain) without shortness of breath, dyspnea, or abnormal imaging. (3) Moderate illness: individuals with a sign of lower respiratory disease by clinical assessment or imaging and oxygen saturation (SaO2) over 93% on room air at sea level. (4) Severe illness: individuals with a respiratory frequency of over 30 breaths per minute, SaO2 up to 93% on room air at sea level, ratio of the arterial partial pressure of oxygen to fraction of inspired oxygen (PaO2/FiO2) below 300, or more than 50% lung infiltrates. (5) Critical illness: individuals with respiratory failure, septic shock, and/or multiple organ dysfunction. The clinical samples were handled and processed in a Bio-Safety Laboratory-2 (BSL-2) at the Faculty of Medicine, Kuwait University, with personal protective equipment and adapted procedures for airborne pathogens by trained personnel.

### Nucleic acid extraction and real-time reverse transcriptase PCR assays for SARS-CoV-2

Total RNA was extracted from 200 μl nasopharyngeal samples using MagNA Pure LC RNA Isolation Kit—High Performance (Roche Applied Science GmbH, Penzberg, Germany) on the MagNA Pure LC 2.0 automated extraction machine (Roche) according to the manufacturer’s instructions. Real-Time RT-PCR was performed to confirm the presence of the virus in the samples using primers and probes corresponding to the SARS-CoV-2 envelope (E), nucleocapsid (N), and RdRP (RNA-dependent RNA polymerase) with SOLIScript one-step multiplex probe kit (ROX; Solis BioDyne, Estonia) according to the manufacturer’s protocol. RT-PCR was performed on the QuantStudio^™^ 5 Real-Time PCR system (Thermo Fisher, United States). All samples were tested for the human RNAseP gene as a housekeeping gene. Extracted nucleic acid from SARS-CoV-2 positive samples was taken for further molecular analysis.

### SARS-CoV-2 whole-genome sequencing using MinION Nanopore technology

The whole genome of SARS-CoV-2 from the clinical samples was sequenced using the Oxford Nanopore sequencing technology (Oxford Nanopore Technologies, Cambridge, United Kingdom) following the ARTIC network protocol. The SARS-CoV-2 positive RNA extracts were reverse-transcripted with LunaScript^™^ RT SuperMix Kit (New England Biolabs, Ipswich, United States). Multiplex PCR with ARTIC Network V3 primer pools, tilling the complete SARS-CoV-2 genome, was performed on cDNA using Q5 Hot Start High-Fidelity DNA polymerase (New England Biolabs, Ipswich, United States). The amplicons were cleaned up with AMPure XP beads (Beckman Coulter Diagnostics, California, United States), and libraries were prepared using the ligation sequencing kit (SQK-LSK109) from Oxford Nanopore Technologies (Oxford, United Kingdom). Then, libraries were quantified using QUBIT 1X dsDNA HS Assay Kit (Invitrogen, Waltham, United States), and 15 ng of each prepared library was loaded into Oxford Nanopore MinION SpotON Flow Cells FLO-MIN106D, R9.4.1 (Oxford Nanopore Technologies, Oxford, United Kingdom (Bull et al., 2020; Pater et al., 2021). The FastQ files generated by the Mk1C device were used for analysis following the ARTIC Network analysis workflow and EP2ME-lab.

### Sequencing of ORF1ab and S regions of SARS-CoV-2 using the Sanger method

The regions in ORF1ab and spike protein that failed to be sequenced by MinION Nanopore technology were amplified and sequenced by specific primers designed using the Primer-BLAST program. The 421 nt and 302 nt fragments covering 19,269-19,689 and 21,139-21,440 regions, respectively, at ORF1ab gene were amplified and sequenced using primers CoV-A/1: 5’- CTTGCCTGGTTGTGATGGTG-3’, CoV-A/2: 5’-TGGTACTTCACCCTGTTGTCC-3’ and Cov-B/1:5’-CTAGCTCTTGGAGGTTCCGTG-3’, CoV-B/2:5’GACATAACAGCAGTACCCCTT-3’. In addition, 591 nt and 595 nt fragments that cover21513-22,103 and 22,020-22,614 regions, respectively, at spike protein gene were amplified and sequenced using primers nCoV-S1F: 5’-CAACAGAGTTGTTATTTCTAGTGATG-3’ and nCoV-S1R: 5’-CTTCAAGGTCCATAAGAAAAGGCT-3’, nCoV-S2F: 5’- TGGAAAGTGAGTTCAGAGTTTATTCT-3’and nCoV-S2R: 5’- TAAACAGATGCAAATCTGGTGGCG-3’. The amplification profile comprised an initial denaturation at 95°C for 5 min, followed by 35 amplification cycles (94°C for 1 min, 65°C for 1 min and 72°C for 2 min), with a final extension at 72°C for 10 min. The amplicons were analyzed by electrophoresis in a 1.5% (W/V) agarose gel. The PCR products were purified with a QIAquick gel extraction kit (Qiagen) according to the manufacturer’s instructions. Subsequently, the purified products were sequenced in the forward and the reverse directions by primers CoV-A/1, CoV-A/2, Cov-B/1, CoV-B/2, nCoV-S1F, nCoV-S1R, nCoV-S2F, and nCoV-S2R. Sequencing was performed using the ABI 3500/3500xL genetic analyzer (PE Applied Biosystems, Inc., Foster City, CA, United States) with the ABI Prism BigDye Terminator Cycle Sequencing Ready Reaction kit (PE Applied Biosystems, Inc., Foster City, CA, United States).

### Bioinformatics analysis

Consensus sequences obtained from the ARTIC analysis pipeline were subjected to multiple sequence alignments using MEGA (MEGA X: Molecular Evolutionary Genetics Analysis across computing platforms; Kumar et al., 2018) and Jalview v2.11.1.4 (Waterhouse et al., 2009). The SARS-CoV-2 reference genome was downloaded from the national centre for biotechnology information (NCBI; NC_045512.2). The resulting consensus sequences were analyzed with sequences identified in the Middle East from late February 2020 to late June 2021 (*n* = 6,169) available in GISAID (Elbe and Buckland-Merrett, 2017a). Using the Augur pipeline (Hadfield et al., 2018a), sequences were aligned to the SARS-CoV-2 reference genome using MAFFT, and time-resolved Maximum–Likelihood phylogenetic trees with 1,000 bootstrap replicates were constructed using IQ-Tree (Nguyen et al., 2015) under the GTR substitution model and visualized with Auspice (Hadfield et al., 2018). The trees were visualized and modified with the Interactive Tree of Life (iTOL) v6 software.^4^ A Heatmap for correlation analysis of the average number of mutations and deletions in each SARS-CoV-2 gene among different variants was performed using GraphPad Prism v9. Clade nomenclature was attained from Nextstrain (Hadfield et al., 2018). Complete genome sequences in GSAID with accession IDs and their Pangolin clade assignation are publicly available in Supplementary Table 1.

## Results

### Demographics and clinical characteristics of the study population

The tested population consisted of 209 SARS-CoV-2–positive patients, of whom 48% were males, and 52% were females. Of the patients, 55% were Kuwaiti, and 45% were non-Kuwaiti. The median age of the patients was 40 years (Table 1). The SARS-CoV-2 infections in the involved patients resulted in a range of clinical outcomes: 28% of patients had an asymptomatic infection, 21% had a symptomatic infection, and did not have information about 51% of the patients (Table 2). Among the symptomatic patients, 27% had mild, 30% had moderate, 30% had severe, 14% fell into critical condition, and 16% died (Table 2). A wide range of symptoms was recorded among the symptomatic patients, with fever (16.4%) and shortness of breath (SOB; 15.8%) being the most common symptoms. However, other symptoms were observed less frequently, including cough, reduced oxygen saturation, and bilateral infiltration of the lungs through X-ray imaging (Table 2).

**Table 1:** Demographic data of the study population (*N* = 209).

Variable	No. of patients (%)
Age, median (IQR), y	40 (22)
Gender	
Male	100 (48)
Female	109 (52)
Nationality	
Kuwaiti	115 (55)
Non-Kuwait	94 (45)

**Table 2:** Clinical outcome and comorbidities of the patients.

Variable	No. of patients (%)
Unknown clinical condition	106 (51)
Asymptomatic	59 (28)
Symptomatic	44 (21)
Mild	12 (27)
Moderate	13 (30)
Severe	13 (30)
Critical	6 (14)
Death	7 (16)
**Symptoms**	
Fever	31 (16.4)
Shortness of breath (SOB)	30 (15.8)
Cough	15 (7.9)
SPO2 88% in RA	12 (6.3)
CXR: B/L infiltrates	12 (6.3)
CXR: B/L peripheral basal haziness	9 (4.8)
Severe cough productive sputum	9 (4.8)
Loss of taste and smell sensation	7 (3.7)
Chest exam: B/L crepitation	6 (3.2)
Generalized fatigue	6 (3.2)
Headache	4 (2.1)
Confusion	4 (2.1)
Vomiting	4 (2.1)
Chest exam: B/L reduced air entry	4 (2.1)
Chest pain	4 (2.1)
Oedema	3 (1.6)
Chest: B/L fair entry	3 (1.6)
Chest infection (COVID pneumonia)	3 (1.6)
Chest infection	3 (1.6)
Chest exam: B/L wheeze.	3 (1.6)
Sore throat	2 (1.1)
Shivering	2 (1.1)
Abdominal pain	2 (1.1)
Nausea	2 (1.1)
CXR: B/L multi-lobe chest inflations	1 (0.5)
Maculopapular skin rash	1 (0.5)
CXR: B/L moderate air-space disease with progressive course	1 (0.5)
Runny nose	1 (0.5)
Dementia	1 (0.5)
Septic shock	1 (0.5)
Chronic obstructive pulmonary disease (COPD)	1 (0.5)
Blood acidosis	1 (0.5)
Ketoacidosis	(0.5)
**Without comorbidity, n (%)**	8 (18.2)
**With comorbidity, n (%)**	36 (81.8)
Diabetes Mellitus (DM)	26 (31.3)
Hypertension (HTN)	21 (25.3)
Ischemic Heart Disease (IHD)	9 (10.8)
Bronchial Asthma (BA)	7 (8.4)
Dyslipidaemia	5 (6.0)
Smokers	5 (6.0)
Hypothyroidism	3 (3.6)
Chronic kidney disease (CKD)	2 (2.4)
End-Stage Renal Disease (ESRD)	1 (1.2)
Multiple congenital anomalies	1 (1.2)
Osteoarthritis	1 (1.2)
Congenital Heart Disease	1 (1.2)

The majority of the symptomatic patients had comorbidities (81.8%), while the rest (18.2%) did not have comorbidities (Table 2). Among patients with comorbidities, 31.1% had diabetes mellitus, 25.3% had hypertension, and 10.8% had ischemic heart disease (Table 2).

### Whole-genome sequencing of SARS-CoV-2 samples

Two hundred sixteen nasopharyngeal swab samples were collected from SARS-CoV-2 positive patients in Kuwait between February 2020 and June 2021; however, we attained 209 high-quality samples for downstream analysis. Using the 29,903 nt SARS-CoV-2 reference genome (NC_045521), we have successfully assembled 209 SARS-CoV-2 consensus genomes. Most of the sequences (96%) were high-quality, with gaps ranging from 300 nt–to 2000 nt (a gap proportion of ~10%). These gaps indicated low-sequence coverage over the 5′ and the 3′ ends of the genomes, a common incidence in worldwide assemblies reported in GISAID. In addition, there were common gaps at the 19,277–19,570 nt positions and the 21,147–21,386 nt positions of the ORF1ab gene and at the 21,513–22,103 nt and 22,020–22,614 nt positions of the S gene in the sequences, which were covered using the Sanger method with customized primers (see the methodology).

### Genomic epidemiology of SARS-CoV-2 infection in Kuwait

**Figure 1:**
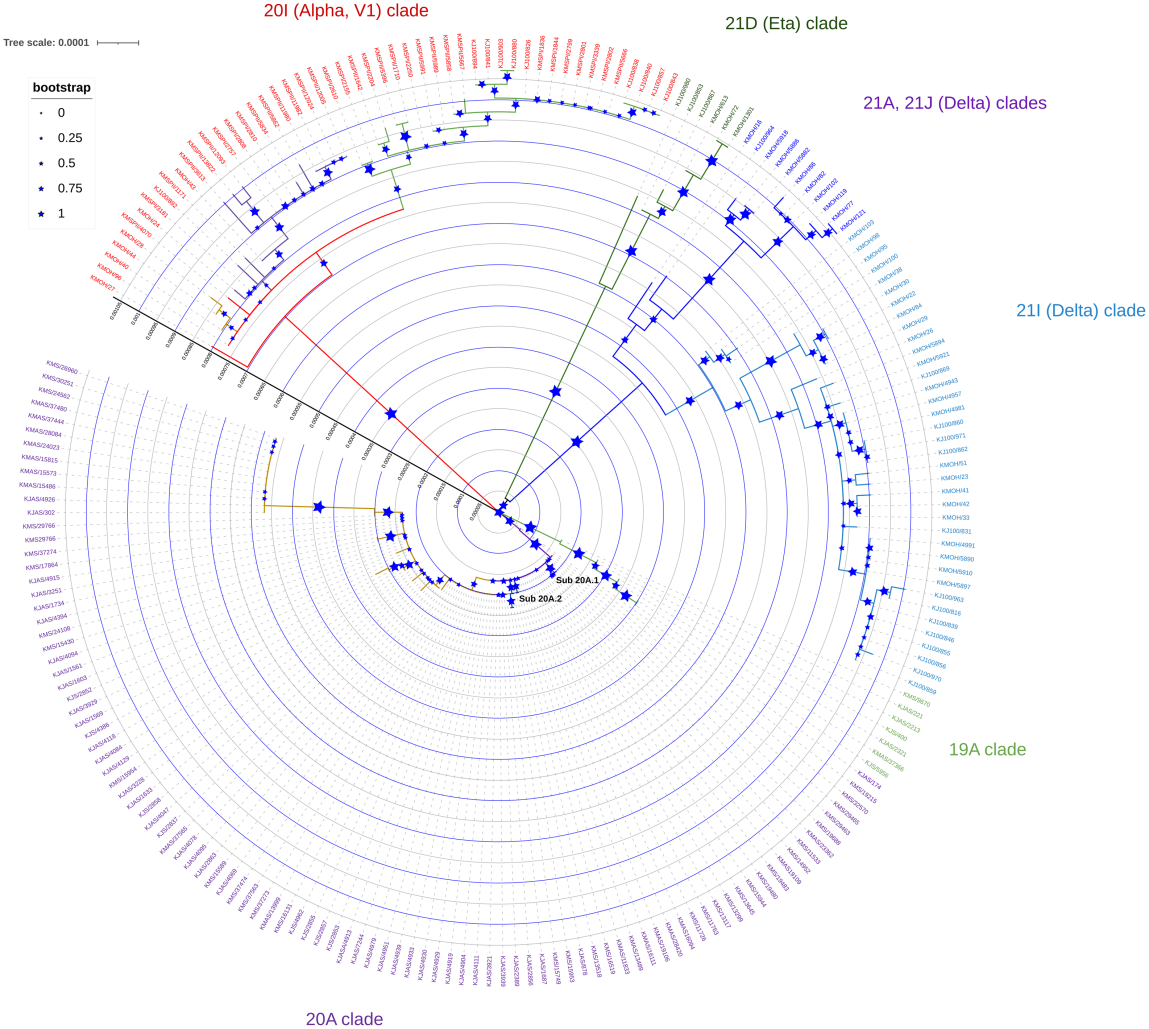
Maximum likelihood tree of 209 SARS-CoV-2 genomes sequenced in Kuwait from February 2020 to June 2021. Each line indicates a sample coloured by the dominant viral clades, annotated with the clade’s definitive genetic variation. Reference strain (NC_045512) is coloured in black.

Phylogenetic analysis of genomes sequenced between February 2020 and June 2021 revealed distinct clustering patterns (Figure 1). Our study’s maximum likelihood phylogenetic tree of the 209 SARS-CoV-2 genomes demonstrated the seven main dominant clades in Kuwait’s viral population. According to the Nextstrain classification, 98 (46.8%) of viral genomes out of the 209 genomes were clustered as clade 20A, while the rest of the genomes sequences were classified in the clades 20I (Alpha, V1; *n* = 50, 24%); 21I (Delta; *n* = 38, 18.2%); 19A (*n* = 8, 3.8%); 21A (Delta; *n* = 7, 3.3%); 21D (Eta; *n* = 6, 3%); and 21 J (Delta; *n* = 3, 1.4%; Figure 1). Detailed analysis showed that clade 20A is further subdivided into two sub-clades, 20A.1 and 20A.2, with bootstrap values of more than 0.75 (Figure 1).

**Figure 2:**
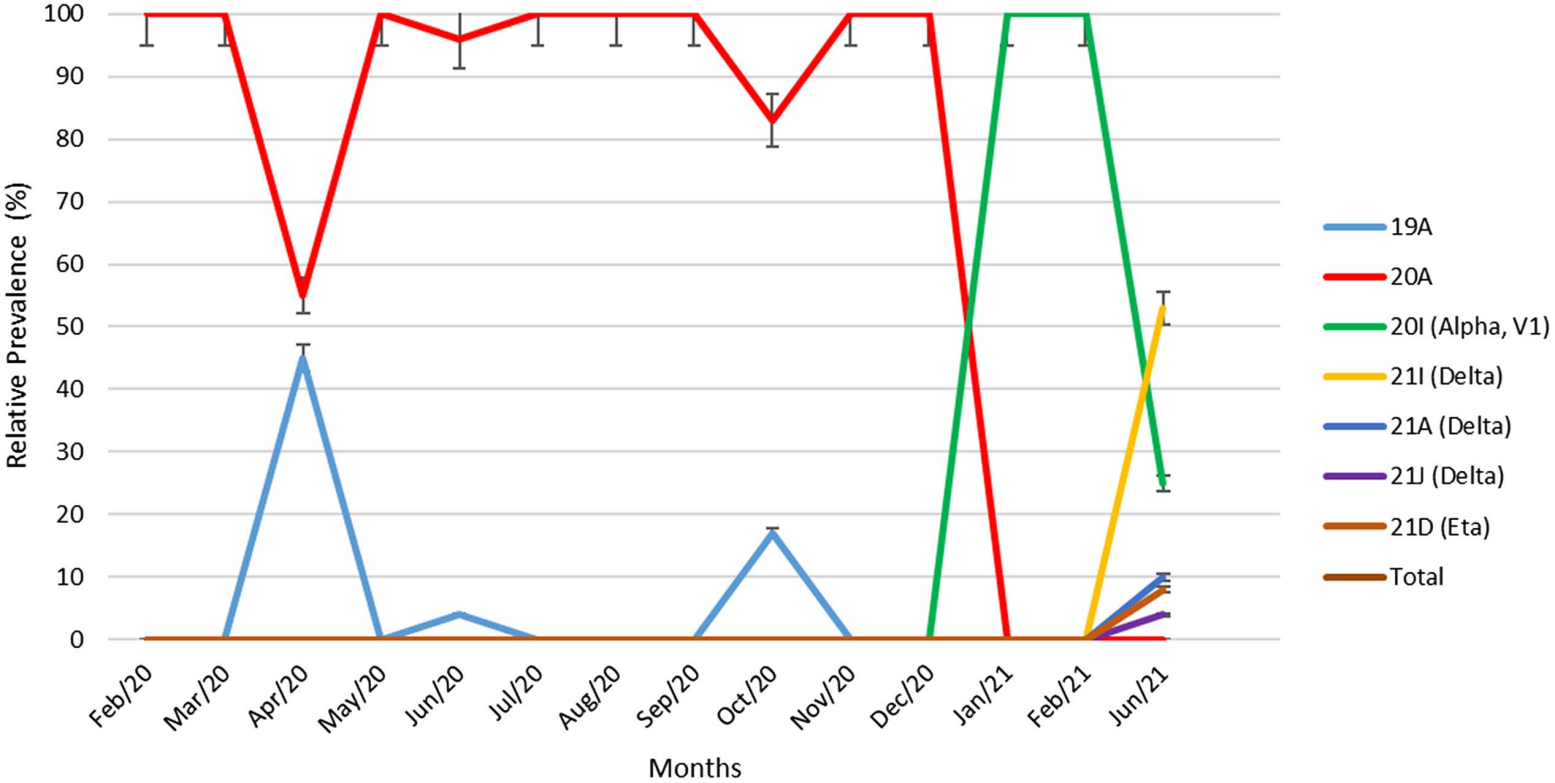
Relative prevalence of SARS-CoV-2 clades circulating in Kuwait from February 2020 to June 2021. Error bars represent a 95% confidence interval.

Figure 2 demonstrates the relative prevalence of SARS-CoV-2 clades circulated in Kuwait from February 2020 to June 2021. The results show that the dominant clades of SARS-CoV-2 from February until January 2021 were 20A and 19A. Clade 20I (alpha, V1) emerged in January 2021 and was the dominant clade until June 2021. In June 2021, the number of genomes belonging to clades 21I, 21A, and 21 J (Delta) increased and dominated, while a few cases of 21D (Eta) emerged. Details on the number of SARS-CoV-2 clades detected in Kuwait each month are provided in Supplementary Table 1.

### Association of Kuwait SARS-CoV-2 genomes with genomes from other middle east countries

**Figure 3:**
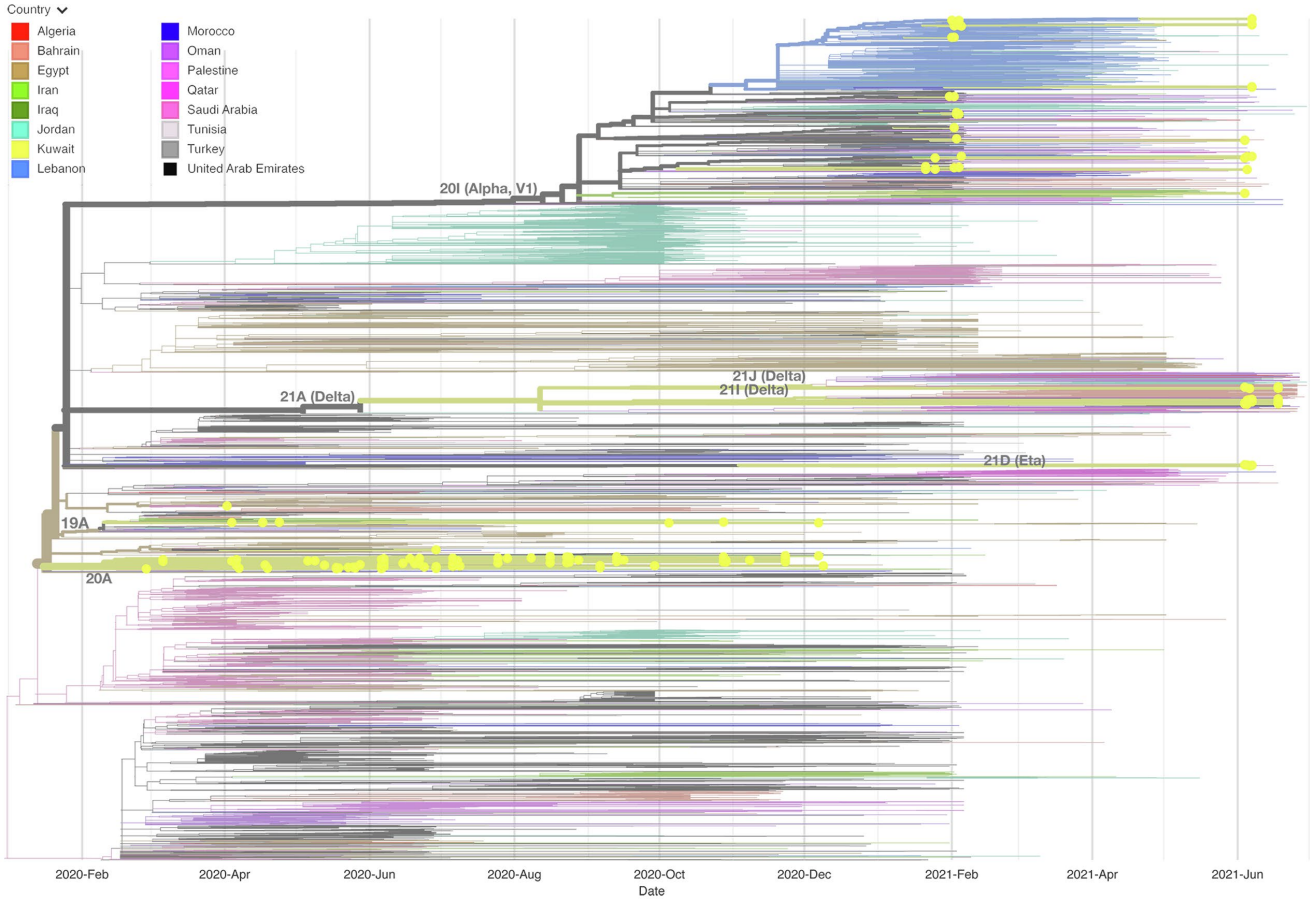
Time-resolved phylogenetic tree representing 6,169 genomes sampled between February 2020 and June 2021. The Whole-genome of 209 SARS-CoV-2 isolates in Kuwait and high coverage genomes from 16 countries in the Middle East from February 2020 to June 2021 available in GSAID were included. The phylogeny was estimated using IQTree under the GTR substitution model and visualized with auspice. The tree was rooted with the reference strain NC_045512.

**Figure 4:**
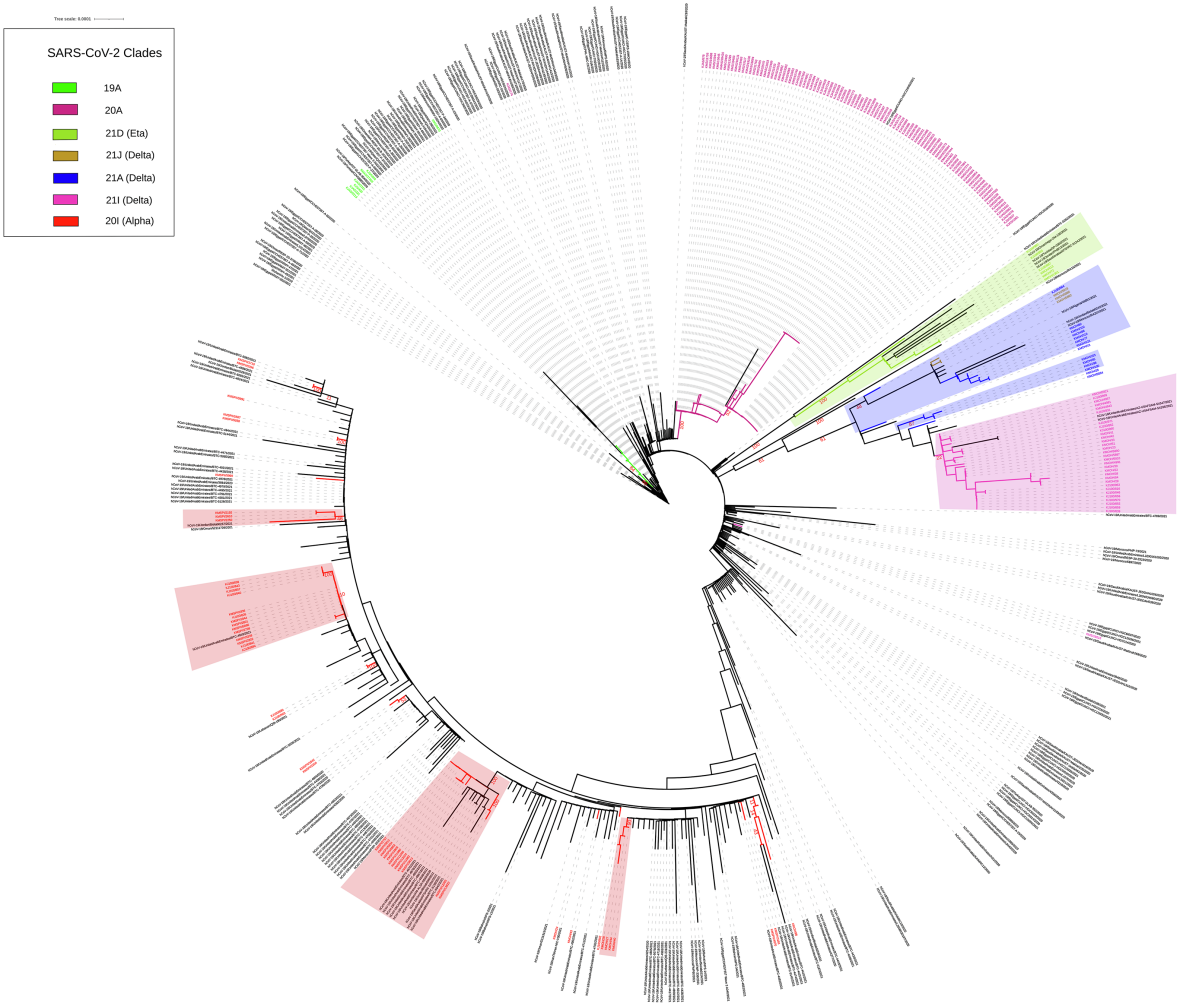
Maximum likelihood IQ TREE of 6,169 SARS-CoV-2 genomes from Middle East countries and Kuwait from February 2020 to June 2021. Kuwaiti clades are in different colours. Bootstrap values are in red.

We built up a time-resolved phylogenetic tree from the genome of 209 SARS-CoV-2 strains in Kuwait and 6,169 high coverage genomes from 16 Middle East countries from February 2020 to June 2021 retrieved from GSAID (Figure 3). Figure 4, on the other hand, shows the viral phylogeny of 6,169 SARS-CoV-2 genomes from Middle East countries and Kuwait from February 2020 to June 2021. Clade 20A constituted 46% of the sampled genomes from Kuwait, and it was the first clade to be detected in Kuwait in February 2020, showing a separate clade of related Kuwaiti strains with a 100% bootstrap value. However, a sub-cluster of Kuwaiti 20A with a bootstrap value of 52% was detected and contained a sequence from Egypt that showed a separate linage (Figure 4). In April 2020, the Kuwaiti clade 19A was detected in Kuwait, and the genomes of the Kuwaiti strains had a bootstrap of 66% and a further sub-cluster with a bootstrap of 84% that contained genomes from Tunisia and Turkey. On the other hand, the 20I (Alpha, V1) genomes were detected in Kuwait in January 2021, and the Kuwaiti genomes showed several clusters with genomes from many countries with variable strength of genetic relationships. A clade of four Kuwaiti 20I (Alpha) strains with 90% bootstrap was detected, while another clade of 12 20I (Alpha) Kuwaiti strains and genomes from UAE, SA, and Jordan showed 100% bootstrap (highlighted in red). However, other cluster had a weak genetic relationship (bootstrap = 10%) with the genome from UAE, while the other had a bootstrap of 46% with the genome from Jordan (Figure 4; highlighted in red). Clade 21A (Delta) was spotted in Kuwait in June 2021, and it was dispersed in three clusters: a cluster with bootstrap 48% that contains seven Kuwaiti 21A (Delta) and sequences from Jordan and Morocco in addition to three sequences of Kuwaiti 21 J (Delta) sequences (highlighted in blue); one sequence alone, and a cluster with bootstrap 87% contained four Kuwaiti 21A (Delta) sequences (highlighted in blue). On the other hand, the Kuwaiti clade 21I (Delta) showed one clade including sequences from UAE with a bootstrap of 22% and other sub-clusters (highlighted in pink). Only a few samples with 21D (Eta) clade were detected in June 2021 and showed a cluster with 100% bootstrap with genomes from UAE, Oman, Tunisia, Jordan, and SA (highlighted in green). A phylogenetic tree comprising all the bootstrap values is provided in Supplementary Figure 1.

### SARS-CoV-2 mutations patterns

We identified positions along the SARS-CoV-2 genome, which were frequently altered across the Kuwaiti sequences when compared with the reference genome. A total of 141 synonymous and missense mutations and deletions were spotted across different gene regions; however, only 124 variant sites with a prevalence of ≥2% were presented (Table 3). Among the 124 variant sites, there were 81 (65.3%) missense mutations, 34 (27.4%) synonymous mutations, and nine (7.3%) deletions. Overall, ORF1ab harbored more mutations and deletions (n = 62, 49.2%) compared to the other 12 gene regions (S: 24, 19.3%; N: 15, 12.1%; ORF8: 8, 6.5%; M: 4, 3.2%; ORF3a: 3, 2.4%; ORF7a: 3, 2.4%; E: 1, 0.8%; ORF6: 1, 0.8%; ORF7b: 1, 0.8%; and ORF9b: 1, 0.8%).

**Table 3:** SARS-CoV-2 frequently observed mutations and deletion in 209 sequences of SARS-CoV-2 isolated in Kuwait.

Serial No.	Position	Gene	NT change	AA change	Type of mutation	Frequency %
1	170	5’UTR	A > G	–	–	6
2	210	5’UTR	G > T	–	–	23
3	228	5’UTR	C > T	–	–	2
4	241	5’UTR	T > C	–	–	3
5	832	Nsp2	C > T	N189N	Synonymous	2
6	913	Nsp2	C > T	S216S	Synonymous	24
7	1,498	Nsp2	C > T	G411G	Synonymous	2
8	1807	Nsp2	A > G	G514G	Synonymous	2
9	1820	Nsp2	G > A	G519S	Missense	2
10	2,453	Nsp2	G > T	L730F	Missense	6
11	2,584	Nsp3	T > A	L733L	Synonymous	6
12	2,659	Nsp3	G > A	K798K	Synonymous	2
13	3,037	Nsp3	T > C	F924F	Synonymous	3
14	3,267	Nsp3	C > T	T1001I	Missense	24
15	4,181	Nsp3	C > T	A1306S	Missense	3
16	5,184	Nsp3	C > T	P1640L	Missense	3
17	5,388	Nsp3	C > A	A1708D	Missense	13
18	5,584	Nsp3	A > G	T1773T	Synonymous	18
19	5,986	Nsp3	C > T	F1907F	Synonymous	24
20	6,285	Nsp3	C > T	T2007I	Missense	2
21	6,402	Nsp3	C > T	P2046L	Missense	5
22	6,449	Nsp3	C > T	L2062F	Missense	16
23	6,954	Nsp3	T > C	I2230T	Missense	22
24	7,124	Nsp3	C > T	P2287S	Missense	4
25	7,360	Nsp3	G > T	M2365I	Missense	2
26	8,296	Nsp4	T > C	Y2677Y	Synonymous	46
27	8,512	Nsp4	A > G	Q2749Q	Synonymous	6
28	8,593	Nsp4	T > C	V2776V	Synonymous	3
29	8,986	Nsp4	C > T	D2907D	Synonymous	4
30	9,053	Nsp4	G > T	V2930L	Missense	4
31	9,514	Nsp4	A > G	L3083L	Synonymous	2
32	9,565	Nsp4	C > T	F3100F	Synonymous	3
33	9,891	Nsp5	C > T	A3209V	Missense	18
34	10,029	Nsp5	C > T	T3255I	Missense	4
35	11,201	Nsp6	A > G	T3646A	Missense	4
36	11,264	Nsp6	T > C	L3667L	Synonymous	2
37	11,288-11,296	Nsp6	Deletion	S3675del-G3676del-F3677del	–	27
38	11,335	Nsp6	G > A	V3690V	Synonymous	10
39	11,418	Nsp6	T > C	V3718A	Missense	16
40	11,514	Nsp6	C > T	T3750I	Missense	18
41	11,758	Nsp7	C > T	P3831P	Synonymous	2
42	12,076	Nsp8	C > T	N3937N	Synonymous	46
43	12,885	Nsp11	C > T	T4207I	Missense	7
44	13,019	Nsp11	C > T	L4252L	Synonymous	18
45	13,618	Nsp12	G > T	D51Y	Missense	6
46	14,373	Nsp12	A > G	A302A	Synonymous	3
47	14,407	Nsp12	C > T	P314L	Missense	97
48	14,676	Nsp12	T > C	P403P	Synonymous	24
49	15,096	Nsp12	T > C	N543N	Synonymous	13
50	15,279	Nsp12	C > T	H604H	Synonymous	24
51	15,451	Nsp12	G > A	G662S	Missense	23
52	16,176	Nsp13	T > C	T903T	Synonymous	24
53	16,466	Nsp13	C > T	P1000L	Missense	23
54	16,653	Nsp13	A > G	K1062K	Synonymous	2
55	17,615	Nsp13	A > G	K1383R	Missense	2
56	17,972	Nsp14	G > A	R1502K	Missense	6
57	18,171	Nsp14	C > T	G1568G	Synonymous	9
58	18,877	Nsp14	C > T	L1804L	Synonymous	46
59	19,220	Nsp14	C > T	A1918V	Missense	4
60	20,724	Nsp16	A > G	L2419L	Synonymous	3
61	21,205	Nsp16	C > T	L2580F	Missense	2
62	21,618	S	C > G	T19R	Missense	23
63	21,762	S	C > T	A67V	Missense	5
64	21,766-21,771	S	Deletion	H69 del/V70 del	–	25
65	21,992-21,994	S	Deletion	Y144 del	–	25
66	22,029-22,034	S	Deletion	E156 del	–	16
67	22,035	S	Deletion	F157 del	–	10
68	22,188	S	C > T	R158G	Missense	3
69	22,227	S	C > T	P209L	Missense	13
70	22,329	S	C > T	A222V	Missense	2
71	22,917	S	T > G	L452R	Missense	23
72	22,995	S	C > A	T478K	Missense	23
73	23,012	S	G > A	E484K	Missense	3
74	23,063	S	A > T	N501Y	Missense	24
75	23,114	S	C > T	L518L	Synonymous	3
76	23,271	S	C > A	A570D	Missense	24
77	23,403	S	A > G	D614G	Missense	97
78	23,593	S	G > C	Q677H	Missense	3
79	23,604	S	C > A	P681H	Missense	47
80	23,709	S	C > T	T716I	Missense	24
81	24,410	S	G > A	D950N	Missense	23
82	24,224	S	T > C	F88L	Missense	2
83	24,506	S	T > G	S982A	Missense	24
84	24,914	S	G > C	D1118H	Missense	19
85	25,096	S	C > T	N1178N	Synonymous	2
86	25,469	ORF3a	C > T	S26L	Missense	23
87	25,563	ORF3a	G > T	Q57H	Missense	46
88	25,785	ORF3a	G > T	W131C	Missense	8
89	26,305	Envelope E	C > T	L21F	Missense	2
90	26,681	Membrane (M)	C > T	F53F	Synonymous	22
91	26,720	Membrane (M)	G > C	V66V	Synonymous	3
92	26,767	Membrane (M)	T > C	I82T	Missense	26
93	27,012	Membrane (M)	C > T	L164L	Synonymous	6
94	27,205-27,207	ORF6	Deletion	M1 del	–	3
95	27,626	ORF7a	G > T	R78L	Missense	2
96	27,638	ORF7a	T > C	V82A	Missense	17
97	27,752	ORF7a	C > T	T120I	Missense	16
98	27,874	ORF7b	C > T	T40I	Missense	5
99	27,972	ORF8	C > T	Q27*	Missense	23
100	28,048	ORF8	G > T	R52I	Missense	23
101	28,077	ORF8	G > T	V62L	Missense	6
102	28,087	ORF8	C > T	A65V	Missense	6
103	28,095	ORF8	A > T	K68*	Missense	13
104	28,111	ORF8	A > G	T73C	Missense	24
105	28,248-28,253	ORF8	Deletion	D119 del	–	23
106	28,248-28,253	ORF8	Deletion	F120 del	–	23
107	28,271	Nucleocapsid (N)	Deletion	M1del		44
108	28,278-28,280	Nucleocapsid (N)	Deletion	S2del		3
109	28,280	Nucleocapsid (N)	G > C	D3L	Missense	24
110	28,281	Nucleocapsid (N)	A > T	D3L	Missense	24
111	28,282	Nucleocapsid (N)	T > A	D3L	Missense	24
112	28,461	Nucleocapsid (N)	A > G	D63G	Missense	23
113	28,690	Nucleocapsid (N)	G > T	L139F	Missense	46
114	28,699	Nucleocapsid (N)	A > G	P142P	Missense	3
115	28,854	Nucleocapsid (N)	C > T	S194L	Missense	34
116	28,881	Nucleocapsid (N)	G > T	R203M	Missense	47
117	28,882	Nucleocapsid (N)	G > A	R203K	Missense	47
118	28,883	Nucleocapsid (N)	G > C	G204R	Missense	24
119	28,887	Nucleocapsid (N)	C > T	T205I	Missense	3
120	28,977	Nucleocapsid (N)	C > T	S235F	Missense	24
121	29,402	Nucleocapsid (N)	G > T	D377Y	Missense	23
122	28,461	ORF10	A > G	T60A	Missense	16
123	29,742	‘3UTR	G > T			28
124	29,764	‘3UTR	G > A			8

123 mutations and deletions along the SARS-CoV-2 genome occurred in > 2% of the 209 Kuwaiti sequences. The list contains each mutation, its position, gene, nucleotide substitution, the originating and altered AA group, % frequency in Kuwaiti sequences, and whether missense or synonymous mutations. Known clade-defining missense mutations are highlighted in grey. Known synonymous mutations are highlighted in light grey. Highly frequent unknown missense mutations are highlighted in green, and highly frequent unknown synonymous mutations are highlighted in blue.

**Figure 5:**
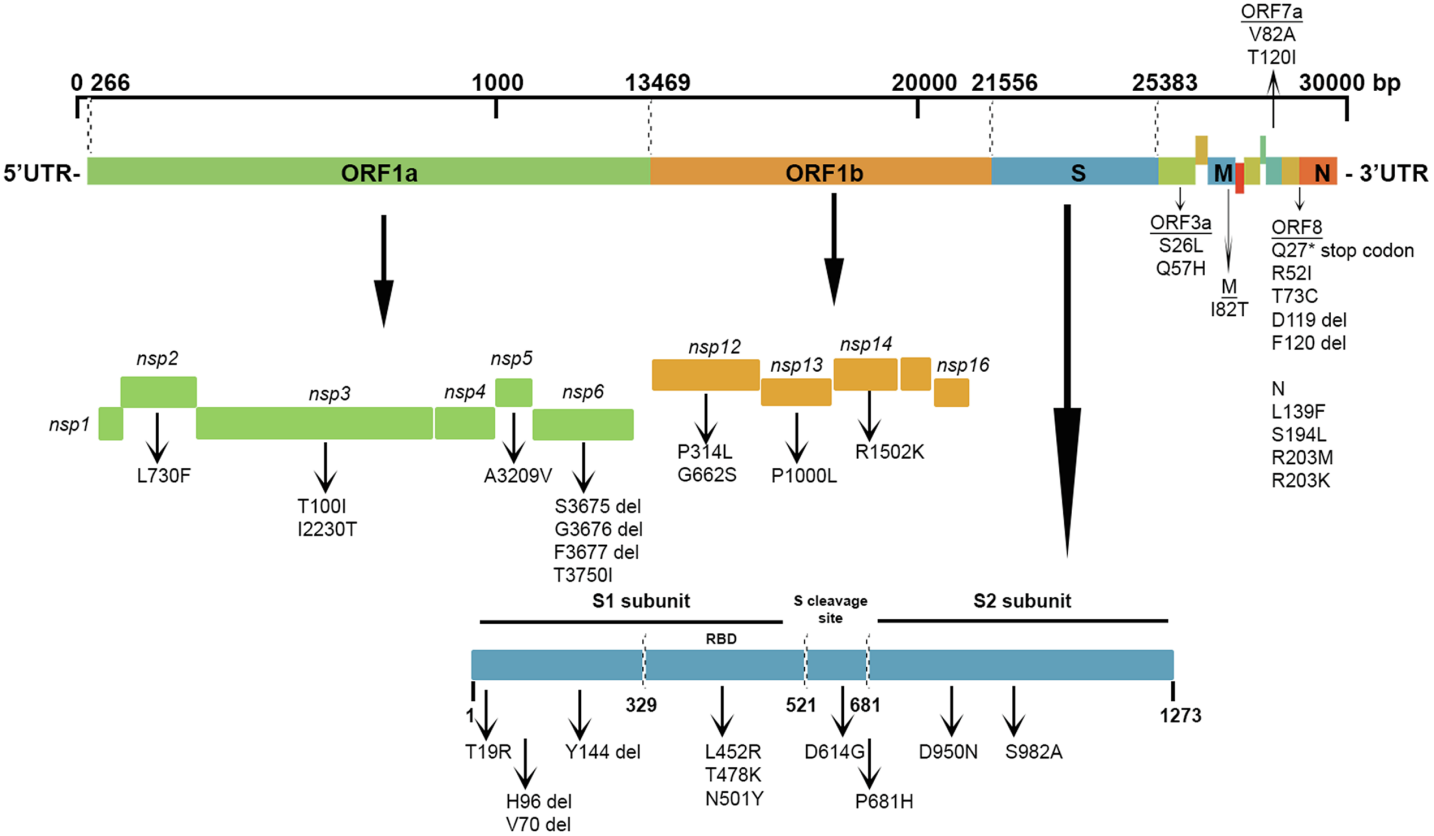
Graphical representation of the SARS-CoV-2 mutation diversity in Kuwait. A scaled genetic map shows the location of genomic mutations and deletions on the complete nucleotide sequence of SARS-CoV-2 isolated in Kuwait. Vertical arrows represent recurrent mutations and deletions. Del, deletions.

The most prevalent missense mutations were P314L (97%) in ORF1b and D614G (97%) in the S glycoprotein regions. Other missense mutations and deletions were detected in over 40% of the sequences: S: P681H (47%), N: R203M/R203K (47%), ORF3a: Q57H (46%), N: L139F (46%), and N: M1 deletion (44%). There were 28 (22.5%) other positions in the Kuwaiti sequences that were altered and were found in >10% of the sequences: ORF1a: L2062F (16%), S: P209L (13%), ORF3a: Q57H (46%), ORF8: K68^*^ (stop codon; 13%), and N: S194L (34%; Table 3). Figure 5 is a graphical representation that shows the location of the most recurrent missense mutations and deletions in each region of the complete nucleotide sequence of SARS-CoV-2 isolated in Kuwait.

### SARS-CoV-2 variants analysis

**Figure 6:**
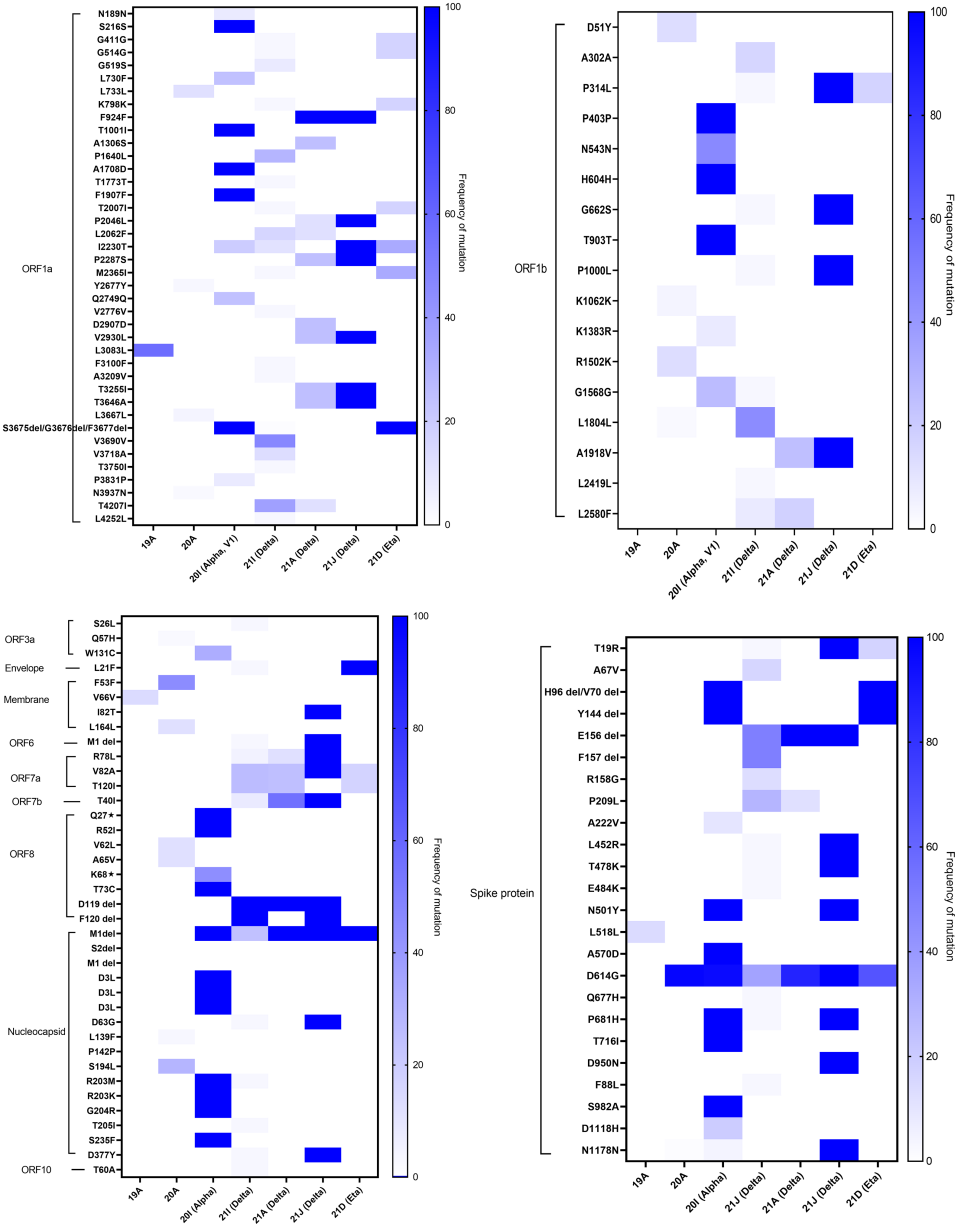
Heatmap demonstrates the percentage of each mutation and deletion in SARS-CoV-2 genes among different variants detected in Kuwait (*n* = 209). The colour scale indicates the significance of the correlation, with blue and white colours indicating the highest and lowest correlation, respectively.

In-depth mutation analysis in each gene region of SARS-CoV-2 was done independently for each of the major variants detected in Kuwait (Figure 6). In ORF1a, seven missense mutations (F924F, P2046L, I2230T, P2287S, V2930L, T3255I, and T3646A) were found in high prevalence (100%) of 21 J (Delta) clade. While, T1001I, A1708D, S3675del, G3676del, and F3677del in the same region were found in 100% of 20I (Alpha, V1) clades. Three deletions (S3675del, G3676del, and F3677del) were found in 100% of 21D (Eta). In ORF1b, 100% of 21 J (Delta) clades had four mutations: P314L, G662S, P1000L, and A1918V. In the spike protein gene, H96del, V70 del, and Y144 del were detected in 100% of 20I (Alpha, V1) and 21 J (Eta) clades; and all 20I (Alpha, V1) clades had four mutations: N501Y, A570D, P681H, and S982A in their genome. However, T19R, L478R, T478K, N501Y, P681H, and D950N mutations in the S region were found in 100 of 21 J (Delta) clades 100% of 21A (Delta) had only one deletion: E156del. Three missense mutations in ORF8 (Q27*, R52I, and T73C), five mutations in the N gene (D3L, R203M, R203K, G204R, and S235F), and one deletion (M1del) in the N gene were found in high prevalence (100%) among 20I (Alpha) clades. On the other hand, the following alterations were found in the highest frequencies (100%) among 21 J (Delta) clades: M: I82T; ORF6: M1del; ORF7a: R78L, V82A; ORF7b: T40I; ORF8: D119 del, and F120 del; N: M1 del, D63G, D377Y, and ORF10: T60A. Also, 100% of 21I (Delta) clades had D119 del and F120 del in the OFRF8 gene. However, 100% of 21A (Delta) clades had D119 del in ORF8 and M1 del in the N gene. Finally, L21F in the E gene and M1 del in the N gene were found in 100% of 21D (Eta) clades. The list of detected known clade-defining mutations in the Kuwaiti SARS-CoV-2 strains is provided in Supplementary Table 2.

## Discussion

On 30 January 2020, the World Health Organization declared the novel coronavirus outbreak in China a public health emergency of international concern (PHEIC), which is the highest alarm level. The outbreak became a worldwide pandemic on 11 March 2020 (Phelan et al., 2020; Timeline of WHO’s Response to COVID-19, 2022). As observed in the rest of the world, Kuwait was introduced in February 2020, with the SARS-CoV-2 pandemic dominated by the Wuhan-HU-1 strain. However, by the first week of March, the virus was reported in many countries, including the Middle East, such as Iran, Pakistan, Afghanistan, Kuwait, Bahrain, Iraq, Oman, and Qatar (Callaway et al., 2020). The first confirmed case of SARS-CoV-2 in Kuwait was announced on 24 February 2020, by a group of travellers from Iran (Madi et al., 2021). The number of cases has increased dramatically since then.

Sequencing for SARS-CoV-2 in Kuwait was established, which will enlarge the perspective to the global data and international genomic associations, comprehend the spread of the virus, and support the epidemiological surveillance for pandemic management. This study describes the genomes of 209 samples of SARS-CoV-2 collected from initial cases early in February 2020 to June 2021.

Commonly, SARS-CoV-2 infection causes flu-like symptoms; however, the symptoms may become severe and lead to ICU admission, whereas others die from the complications (Ortiz-Prado et al., 2020). Our study showed that fever (16.4%) and SOB (15.8%) were frequent events among the admitted symptomatic patients, while other symptoms were less frequent; similar results were reported in other studies (Cui et al., 2019, 2021). COVID-19 patients with diabetes mellitus, hypertension, chronic obstructive pulmonary disease (COPD), cardiovascular diseases (CVD), malignancies, HIV, and other comorbidities develop a life-threatening outcome (Ejaz et al., 2020). Similarly, our results showed that patients with diabetes mellitus (31.3%), hypertension (25.3%) and ischemic heart disease (10.8%) were the most common comorbidities in our group study.

Our analysis of the 209 viral genomes collected in Kuwait suggests that during February 2020 and June 2021, there were multiple clades of SARS-CoV-2. The seven dominant global clades of SARS-CoV-2 were commonly present in Kuwait. The highest prevalence of the 20A clade, followed by the 20I (Alpha, V1) clade, indicates the effects of a larger originator population size or positive selection. Among the main clades, we have identified some sub-clades in Kuwait indicating the inter-clade variations, which might be potentially neutral in their effect.

At the beginning of the pandemic, the circulated viruses were closely related to the Wuhan-Hu-1 strain; however, a variant with D614G substitution in the spike protein gene emerged with increased prevalence. It was shown *in vitro* that due to the higher affinity for ACE2, patients infected with this virus showed higher infectious titters and shed more viral nucleic acid than the wild-type (D614) Wuhan-Hu-1 strain (Korber et al., 2020). Moreover, from February to December 2020, more than 80 lineages (according to PANGO nomenclature) of SARS-CoV-2 were identified (GISAID; Hadfield et al., 2018). Among these lineages, B.1 lineage (includes 20A clade) dominated, accounting for 17% of COVID-19 cases, followed by B.1.1 (13%; Rambaut et al., 2016, 2020; Elbe and Buckland-Merrett, 2017). Early cases were detected in Kuwait in February, and the dominant clade in this period was 20A, found in 46.8% of the Kuwaiti sequences. However, from April 2020, clade 19A was found in 3.3% of the Kuwaiti sequences, and our results showed genome clustering with 19A genomes from Tunisia and Turkey. Starting from September 2020, several genetic clades emerged (B.1.1.7/20I [Alpha]), South Africa (B.1.351/20H [Beta, V2), and Brazil (P.1/20 J [Gamma]), India (B.1.617/21A, 21I, 21 J [Delta]). The CDC classified these five identified variants, based on their impact on transmission and neutralization, as variants of concern (VOC). Of note, the 20I (Alpha, V1) clade was first detected in Kuwait in January 2021, shortly after their emergence in the United Kingdom with travellers from different countries; however, the Kuwaiti genomes clustered with the 20I (Alpha, V1) clade genomes from many Middle East countries such as UAE, SA, and Jordan. In June 2021, 21A, 21I, 21 J (Delta) clades, and less commonly 21D (Eta) clades emerged in Kuwait, and our results showed that their genomes clustered with the genomes of clades from many Middle East countries.

To further discover patterns in viral evolution, we recognized positions along the SARS-CoV-2 genome that were frequently altered across the Kuwaiti sequences. Our data showed more missense mutations than synonymous mutations, and the ORF1ab gene region had more mutations and deletions than other genes. The most prevalent missense mutations were P314L (97%) in ORF1b and D614G (97%) in the S glycoprotein regions. The prevalence of these two mutations increased with the progress of the pandemic, and they have been spotted in all sequences from April 2020 onwards (Muhammad Ansori et al., 2020). It was shown that the D614G mutation is in linkage disequilibrium with the ORF1b gene P314L substitution, and in almost all cases, ORF1b P314L and Spike D614G variants co-occur (Korber et al., 2020; Mak et al., 2020; Yin, 2020; Haddad et al., 2021; Nidom et al., 2021). Besides the clade-defining mutations, we identified 28 unique mutations in the Kuwaiti sequences. Among these mutations, Q57H in ORF3a was found in 46% of the Kuwaiti sequences. This mutation accrued in 7.88% of all samples in 191 countries (Chu et al., 2021; Wang et al., 2021). The first strain collected in January 2020 with this mutation was hCoV-19/Argentina/PAIS-A1026/2020. However, the amino acid change most recently occurred in strain hCoV-19/Belgium/CHUNamur13897030/2021, collected in December 2021 (Elbe and Buckland-Merrett, 2017). The S194L mutation in the N region was found in 34% of the Kuwaiti sequences; however, this mutation was found in low frequency (0.36%) in 108 countries (Barona-Gómez et al., 2021; Sarkar et al., 2021). The first strain with this mutation, collected in March 2020, was hCoV-19/France/IDF_PSL_178/2020. The mutation most recently occurred in strain hCoV-19/Czech Republic/FNO-211885/2021, collected in December 2021. It is worth mentioning that the L2062F mutation in the ORF1a region is one of the rarest mutations detected in all clades; it is found at a frequency of 0.001%^5^; however, it was found in 16% of the Kuwaiti sequences. Also, the K68* (stop) mutation in the ORF8 region was found in 13% of the Kuwaiti sequences; however, globally, it was found in 5.8% of all samples in 160 countries. It is suggested that the presence of this mutation approves that ORF8 is prone to accumulate non-sense variants and the clades transmit effectively without expression of ORF8 (Farkas et al., 2021; Lobiuc et al., 2021). The first strain with this amino acid change, collected in March 2020, was hCoV-19/USA/IL-NM029/2020. The amino acid change most recently occurred in strain hCoV-19/Netherlands/ZH-RIVM-79120/2021, collected in December 2021. Interestingly, the P209L mutation in the S region is a novel mutation found in 13% of the Kuwaiti sequences, precisely in 21 J and 21A (Delta) clades that were not detected elsewhere. The P209L mutation is located in the S1 subunit of the S protein. The S1 protein consists of an N-terminal domain (NTD) and a C-terminal domain (CTD), which recognizes and binds to hACE2 receptors; therefore, it has a vital role in viral infection and pathogenesis (Cosar et al., 2021).

Our data showed that 20I (Alpha, V1) and 21 J (Delta) clades harbored the highest number of mutations in all the regions of the virus. The data also showed that specific mutations were found in all the clade sequences. It is worth mentioning that amongst all alterations, the M1 deletion in the N region was a common alteration found in 20I (Alpha, V1), 21I (Delta), 21A (Delta), 21 J (Delta), and 21D (Eta).

Further investigations are required to understand the molecular and pathophysiological impact of different alterations in the genome of SARS-CoV-2 clades circulated in Kuwait. In addition, further studies are required to predict the influence of accumulated mutations on viral infectivity, immune response, or disease severity. This information is essential to evaluate Kuwait’s virus population structure and infection dynamics.

## Conclusion

This is the first descriptive molecular epidemiology study of SARS-CoV-2 in Kuwait, where the whole-genome sequencing of 209 samples of SARS-CoV-2 coupled with a detailed analysis of the variations in the genome was performed. This has permitted us to explore the genetic variation of the virus and enabled genotype tracking and identifying the mutations present in circulating strains. These results provide baseline information to which forthcoming genomes can be compared to study the evolution of SARS-CoV-2.

## Data availability statement

The datasets presented in this study can be found in online repositories. The names of the repository/repositories and accession number(s) can be found at: GISAID - EPI_ISL_8997316 – 8997524.

## Ethics statement

The studies involving human participants were reviewed and approved by Health Sciences Center (HSC) and the Ministry of Health, Kuwait (No: 2020/1461). Written informed consent for participation was not required for this study in accordance with the national legislation and the institutional requirements.

## Author contributions

NM and HS: conceptualization and experimental design. MA, MS, EA, and AA-M: provided clinical samples and clinical data. HS: investigated and performed experiments. NM: supervision, analyzed the data, wrote and edited the manuscript. FB: contributed to bioinformatics. AM, WC, and HS reviewed the manuscript. All authors discussed the results and contributed to the final manuscript.

## Funding

This project was funded by the Kuwait Foundation for the Advancement of Sciences (KFAS), grant number PN20-13MC-09.

## Conflict of interest

The authors declare that the research was conducted in the absence of any commercial or financial relationships that could be construed as a potential conflict of interest.

## Publisher’s note

All claims expressed in this article are solely those of the authors and do not necessarily represent those of their affiliated organizations, or those of the publisher, the editors and the reviewers. Any product that may be evaluated in this article, or claim that may be made by its manufacturer, is not guaranteed or endorsed by the publisher.

## Footnotes

^1^ https://www.worldometers.info/coronavirus/

^2^ https://outbreak.info/

^3^ https://covid19.who.int/region/emro/country/kw

^4^ https://www.graphpad.com/

^5^ http://cov-glue.cvr.gla.ac.uk/

## Supplementary material

The Supplementary materials for this article can be found online at: https://www.frontiersin.org/articles/10.3389/fmicb.2022.858770/full#supplementary-material

SUPPLEMENTARY FIGURE 1 Maximum likelihood IQ TREE of 6169 SARS-CoV-2 genomes with exact bootstrap values from Middle East countries and Kuwait from February 2020 to June 2021. Kuwaiti clades are in different colours.
