# Supplementary material for: Molecular epidemiology and genetic characterization of SARS-CoV-2 in Kuwait: A descriptive study
Source: Front Microbiol. 2022 Aug 26;13:858770. doi: 10.3389/fmicb.2022.858770 (PMC9459148; doi:10.3389/fmicb.2022.858770)
Supplement: Supplementary file 2 [file Table_1.DOCX]

**Supplement I**

The number of SARS-CoV-2 clades detected in Kuwait every month from February 2020 to June 2021.

| **Date** | **19A** | **20A** | **20I (Alpha, V1)** | **21I (Delta)** | **21A (Delta)** | **21J (Delta)** | **21D (Eta)** | **Total No.** |
| --- | --- | --- | --- | --- | --- | --- | --- | --- |
| **Feb-20** | 0 | 1 | 0 | 0 | 0 | 0 | 0 | 1 |
| **Mar-20** | 0 | 3 | 0 | 0 | 0 | 0 | 0 | 3 |
| **Apr-20** | 5 | 6 | 0 | 0 | 0 | 0 | 0 | 11 |
| **May-20** | 0 | 11 | 0 | 0 | 0 | 0 | 0 | 11 |
| **Jun-20** | 1 | 25 | 0 | 0 | 0 | 0 | 0 | 26 |
| **Jul-20** | 0 | 9 | 0 | 0 | 0 | 0 | 0 | 9 |
| **Aug-20** | 0 | 17 | 0 | 0 | 0 | 0 | 0 | 17 |
| **Sep-20** | 0 | 6 | 0 | 0 | 0 | 0 | 0 | 6 |
| **Oct-20** | 1 | 5 | 0 | 0 | 0 | 0 | 0 | 6 |
| **Nov-20** | 0 | 11 | 0 | 0 | 0 | 0 | 0 | 11 |
| **Dec-20** | 0 | 4 | 0 | 0 | 0 | 0 | 0 | 4 |
| **Jan-21** | 0 | 0 | 9 | 0 | 0 | 0 | 0 | 9 |
| **Feb-21** | 0 | 0 | 23 | 0 | 0 | 0 | 0 | 23 |
| **Jun-21** | 0 | 0 | 18 | 38 | 7 | 3 | 6 | 72 |
| **Total** | 7 | 98 | 50 | 38 | 7 | 3 | 6 | 209 |
