# Supplementary material for: Molecular epidemiology and genetic characterization of SARS-CoV-2 in Kuwait: A descriptive study
Source: Front Microbiol. 2022 Aug 26;13:858770. doi: 10.3389/fmicb.2022.858770 (PMC9459148; doi:10.3389/fmicb.2022.858770)
Supplement: Supplementary file 3 [file Table_2.DOCX]

**Supplement III**

List of known clade-defining muatations detected in Kuwaiti SARS-CoV-2 strains.

| **19A clade** | **20A clade** | **20I (lpha, V1)** | **21I (Delta)** | **21A (Delta)** | **21J (Delta)** | **21D (Eta)** |
| --- | --- | --- | --- | --- | --- | --- |
| - | S: H69- | S: H69- | S: T19R | S: E156- | S: T19R | S: H69- |
| - | S:V70- | S:V70- | S:E156- | S:D614G | S:E156- | S:V70- |
| - | S: H69- | S: Y144- | S: F157- | ORF7a: V82A | S: L452R | S: Y144- |
| - | S:Y144- | S:N501Y | S:R158G | ORF7a:T120I | S:T478K | S:D614G |
| - | S:E484K | S:A570D | S:L452R | ORF8:D119- | S:D614G | ORF1a:T2007I |
| - | S:D614G | S:D614G | S:T478K |  | S:P681R | ORF1a:S3675- |
| - | S:P681H | S:P681H | S:D614G |  | S:D950N | ORF1a:G3676- |
| - | ORF1a:S3675- | S:T716I | ORF1a:P1640L |  | ORF1a:V2930L | ORF1a:F3677- |
| - | ORF1a:G3676- | S:D1118H | ORF1a:A3209V |  | ORF1a:T3255I |  |
| - | ORF1a:F3677- | ORF1a:T1001I | ORF1a:V3718A |  | ORF1a:T3646A |  |
| - | ORF1b:P314L | ORF1a:A1708D | ORF1a:T3750I |  | ORF1b:P314L |  |
|  |  | ORF1a:I2230T | ORF1b:P314L |  | ORF1b:G662S |  |
|  |  | ORF1a:S3675- | ORF1b:G662S |  | ORF1b:P1000L |  |
|  |  | ORF1a:G3676- | ORF1b:P1000L |  | ORF1b:A1918V |  |
|  |  | ORF1a:F3677- | N:D63G |  | M:I82T |  |
|  |  | N:D3L | N:R203M |  | N:D63G |  |
|  |  | N:R203K | N:D377Y |  | ORF1a:V2930L |  |
|  |  | N:G204R | ORF3a:S26L |  | ORF1a:T3255I |  |
|  |  | N:S235F | ORF7a:V82A |  | N:D377Y |  |
|  |  | ORF8:Q27* | ORF7a:T120I |  | ORF7a:V82A |  |
|  |  | ORF8:R52I | ORF8:D119- |  | ORF7b:T40I |  |
|  |  | ORF8:Y73C | ORF8:F120- |  | ORF8:D119- |  |
|  |  |  |  |  | ORF8:F120- |  |
